# Multi-Target Drugs for Blood Cancer in the Elderly: Implications of Damage and Repair in the Cardiovascular Toxicity

**DOI:** 10.3389/fphys.2021.792751

**Published:** 2021-12-07

**Authors:** Rosalinda Madonna

**Affiliations:** ^1^Cardiology Division, University of Pisa, Pisa, Italy; ^2^Department of Internal Medicine, McGovern School of Medicine, The University of Texas Health Science Center at Houston, Houston, TX, United States

**Keywords:** tyrosine kinase inhibitor, proteasome inhibitor, vascular toxicity, cardiotoxicity, NOTCH-1

Hematological malignancies in the elderly with cardiovascular comorbidities are difficult to treat due to the increased cardiovascular toxicity of anticancer therapies. The difficulty of balancing efficacy and tolerability in this group of patients often leads the clinician to suspend or reduce the dosage of drugs, facilitating the relapse of the disease.

The identification of cardiac and vascular progenitor cells in the elderly as target cells may allow the development of new strategies for cardiovascular protection from anticancer drugs. In this commentary, we will focus on cardiovascular toxicity due to some very effective target therapies that have seen the greatest increase for the treatment of hematological malignancies relevant to the elderly—tyrosine kinase inhibitors and proteasome inhibitors.

## Tyrosine Kinase Inhibitors and Cardiovascular Toxicity

Chronic myeloid leukemia is a clonal myeloproliferative disease of primitive hematopoietic progenitor cells (Faderl et al., 1999). It often affects advanced age and 25% of cases are over 60 years old. Goals of chronic myeloid leukemia treatment are: normalization of hematopoiesis (complete hematologic response), elimination of the Ph chromosome from bone marrow cells (complete cytogenetic response) and reduction of Abelson- Breakpoint Cluster Region (BCR-ABL) transcript levels from peripheral blood and bone marrow (major molecular response) (Sawyers, 1999). BCR–ABL fusion gene comes from a reciprocal translocation (Melero-Martin et al., 2008; van de Donk et al., 2021) (q34; q11), known as the Ph chromosome, which results in the formation of a mutated tyrosine kinase protein, called BCR/ABL. The mutated tyrosin kinase protein is constitutively expressed and leads to de-regulated cell proliferation and tumor formation (Blume-Jensen and Hunter, 2001).

**Figure 1:**
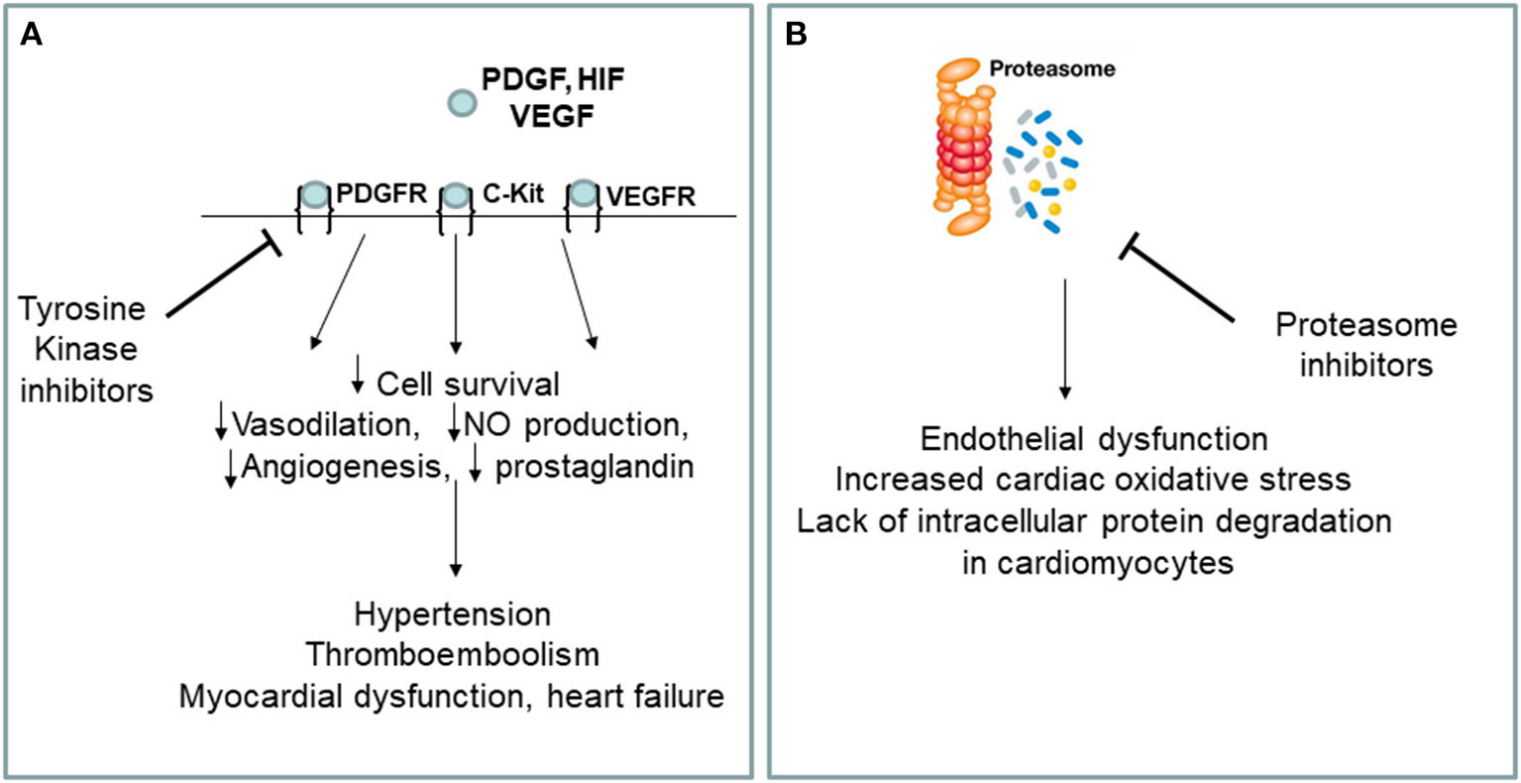
**(A,B)** Putative mechanisms of cardiovascular toxicity induced by tyrosine kinase and proteasome inhibitors. VEGF, vascular endothelial growth factor, VEGFR, vascular endothelial growth factor receptor; PDGF, platelet derived growth factor; PDGFR, platelet derived growth factor receptor; HIF, hipoxia inducible factor; NO, nitric oxide; c-kit, stem cell factor receptor.

The best drugs for the treatment of chronic myeloid leukemia are BCR-ABL tyrosine kinase (TKIs) inhibitors, including Imatinib mesylate, Gefitinib, Erlotinib, Lapatinib, Canertinib, Semaxinib, Vatalanib, Sorafenib, Leflunomide, Nilotinib, Desatinib and Ponatinib. Since the current diagnosis of cardiotoxicity is still based on heart failure symptoms onset or left ventricular ejection fraction (LVEF) drop, and because of the interobserver variability of LVEF measurement, the incidence of cardiotoxicity may vary between anti-cancer drugs and within the same class of drugs, depending on the type of detection system used to formulate the diagnosis. Currently, the cardiotoxicity induced by TKIs in terms of left ventricular dysfunction is around 2.7–19% for sunitinib, between 2 and 4% for desatinib and between 0.2 and 1.5% for imatinib. No randomized clinical trials have been conducted comparing the incidence of different clinical setting of cardiotoxicity for each TKIs. Ponatinib, a third generation TKI, allows to inhibit the T315I mutation of BCR/ABL (Haberbosch et al., 2016). However, recent studies have shown a significant increase in the incidence of cardiotoxicity and vascular adverse events (VAEs) in patients treated with TKIs (Figure 1A), especially increased arterial blood pressure, venous thrombosis, and progressive atherosclerosis with coronary artery disease (CAD) and peripheral arterial obstructive disease (PAOD) (Jain et al., 2015; Moslehi and Deininger, 2015; Caldemeyer et al., 2016; Tournaire et al., 2016). CAD and PAOD are closely associated with endothelial damage, which is the result of the imbalance between vascular damage and vascular repair. TKI-induced VAEs are not a rare phenomenon and may occur in up to 20–42% of patients (Moslehi and Deininger, 2015). The exact etiology of cardiotoxicity and VAEs is not clear, especially with regards to the responsibility of the drug in the onset of them. One hypothesis is that the vascular and cardiac toxicity associated with TKIs could be the result of their broad-spectrum inhibition of kinases in vascular endothelial cells and its progenitor cells and cardiac progenitor cells.

## Proteasome Inhibitors and Cardiovascular Toxicity

Among the various types of blood cancer that affect the elderly, multiple myeloma represents an important clinical entity for which there is currently no valid cure. Multiple myeloma is a clonal myeloproliferative disease of primitive hematopoietic progenitor cells (van de Donk et al., 2021). Normalization of hematopoiesis with complete hematologic response is the goals of multiple myeloma treatment. The best drugs for the multiple myeloma treatment are proteasome inhibitors (PIs) including carfilzomib and bortezomib. Carfilzomib, an epoxyketone PI that binds selectively and irreversibly to the constitutive proteasome and immunoproteasome, has previously shown an encouraging overall response rate of 25.8% in patients with relapsed and refractory multiple myeloma who harbored high-risk cytogenetic abnormalities (Ito, 2012). Carfilzomib is approved in the United States for use in combination with dexamethasone and as a single agent for the treatment of patients with relapsed or refractory multiple myeloma (Dimopoulos et al., 2020). Recent studies have shown a significant increase in the incidence of cardiovascular events in patients treated with PIs, especially arrhythmia, pulmonary edema and heart failure (Waxman et al., 2018) (Figure 1B). Of the proteasome inhibitors used in clinical practice, carfilzomib is the most strongly associated with cardiotoxicity for which the incidence of left ventricular dysfunction ranges between 11 and 25%, while for bortezomib it ranges between 2 and 5%. Overall, 22.1% of patients reported cardiac-related adverse events (Waxman et al., 2018). Studies on the mechanisms of PIs-induced cardiovascular events are not clear, especially for what concerning the drug responsibility in the onset of heart failure. The toxicity that has been associated with PIs could be a result of its broad-spectrum inhibition of the proteasome which could affect cancer cells, as well as non-cancer cells circulating in the blood. There are several hypothesis about mechanisms of cardiotoxicity induced by PIs, including the up-regulation of NF-κB signaling in cardiomyocytes and vascular smooth muscle endothelium, leading to apoptosis. In addition, it seems that PIs downregulate autophagy and nitric oxide homeostasis (Wu et al., 2020).

## The Imbalance of Cardiovascular Damage and Repair as Target for Cardiovascular Toxicity

Endothelial dysfunction is one of the earliest steps of atherosclerosis. TKIs have been shown to promote atherosclerosis by inducing the expression of pro-atherogenic adhesion molecules (CAM) on endothelial cells, including ICAM-1 (CD54), VCAM-1 (CD106) and E-Selectin (CD62E) (Majewska et al., 1997). Since TKIs have broad-spectrum inhibitory effects of tyrosine kinases, in addition to inhibiting BCR-ABL TKIs also inhibit the stem cell factor receptor(c-kit), the platelet-derived growth factor receptor (PDGFR) (Papadopoulos and Lennartsson, 2018) and the type 2 of vascular endothelial growth factor receptor (VEGF) (Zschabitz and Grullich, 2018). Thus, inhibition of tyrosine kinase underline the therapeutic effects of this drug, as well as its vascular adverse events. We have shown that ponatinib inhibits endothelial and endothelial cell progenitor (EPC) survival, reduces angiogenesis and induces endothelial senescence and apoptosis *via* the Notch-1 pathway (Madonna et al., 2020). Hyperactivation of Notch-1 in the vessels can lead to abnormal vascular development and vascular dysfunction (Madonna et al., 2020). By hyperactivating Notch-1 in the vessels, ponatinib exerts an “on-target off tumor effect,” which leads to deleterious effects and may explain the drug's vasculotoxicity. Selective blockade of Notch-1 prevented ponatinib-induced vascular toxicity (Madonna et al., 2020). In a murine model of ponatinib-induced cardiotoxicity (Madonna et al., 2021) we have recently shown sex-related different susceptibility to ponatinib-induced cardiotoxicity, higher in male than female mice, as revealed by: (1) higher numbers of TUNEL-positive cells, (2) higher percentage of SA β-gal-positive senescent cardiac areas, (3) lower expression of the survival marker Bmi1, (4) more pronounced disruption of myofiber structure, (5) predicted downstream activation of cell death and inhibition of cell survival at proteomic analysis, (6) more pronounced left ventricular systolic dysfunction in male mice than in female counterparts (Madonna et al., 2021). Both males and females showed decreased vessel density and downregulation of angiogenesis markers in response to ponatinib treatment in the absence of statistically significant inter-sex differences. Furthermore, at baseline and regardless of ponatinib treatment, female mice exhibited greater cardiac fibrosis, associated with greater diastolic dysfunction (Madonna et al., 2021). We also demonstrated that ponatinib exerts its cardiotoxic effects through the Notch1 pathway. Notch-1 silencing prevented ponatinib-induced cardiac apoptosis and senescence, as well as vascular toxicity (Madonna et al., 2021).

Stem/progenitor cells from both adult and embryonic tissues have been investigated experimentally for their ability to repair and regenerate cardiovascular tissues. In particular, vascular progenitor cells residing in the vessel wall or circulating in the blood, have attracted much attention as a mechanism for repairing vascular damage and for replacing exfoliated endothelial cells (Hill et al., 2003; Ingram et al., 2004; Madonna and Caterina, 2015). Functionally, vascular progenitor cells play an important role in the regeneration of damaged tissues by repairing the denuded endothelium in the injured vessel (Melero-Martin et al., 2008). Mostly important, vascular progenitor cells promote cardioprotection and cytoprotection by releasing paracrine factors such as VEGF, granulocyte-colony stimulating factor (G-CSF), stem cell derived factor (SDF)-1α and insulin growth factor (IGF)-1 (Chistiakov et al., 2016). Along with vascular progenitor cells, cardiosphere are believed to have regenerative and reparative properties for the damaged heart. Cardiospheres (CSp) are a heterogeneous population clinically candidate for cell therapy, containing cardiac progenitors and cardiac stromal cells (CSC), the latter with support and paracrine functions (Makkar et al., 2020). Cross-talk between cardiomyocytes and CSC plays a critical role in maintaining normal cardiac function and contributes to cardiac remodeling after ischemia (Plotnikov et al., 2008). This can be hampered by aging and accelerated by pharmacological insults such as antineoplastic drugs, which can impair cardiac repair, favoring heart failure (Martini et al., 2019).

**Figure 2:**
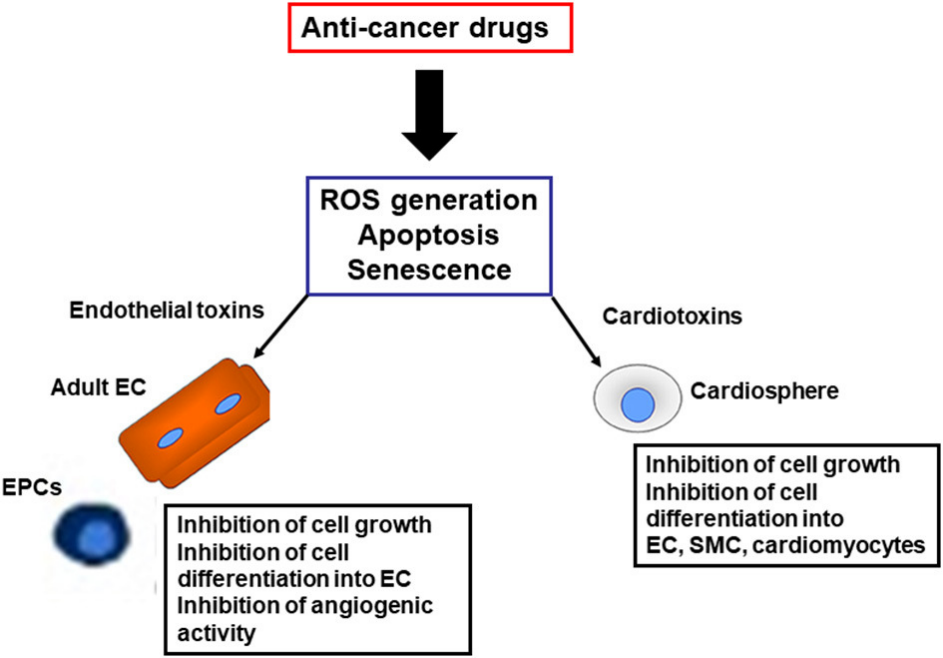
Harmful effects induced by anti-cancer drugs on reparative and regenerative cardiovascular cells. EC, endothelial cells; SMC, smooth muscle cells; EPC, endothelial progenitor cells; ROS, reactive oxygen species.

One hypothesis is that cardiovascular complications of TKIs (Madonna et al., 2020) and PIs may reflect a “stem cell vasculopathy and cardiotoxicity,” in which the defective reparative and regenerative cellular compartment is unable to regenerate dying endothelial cells and cardiomyocytes, contributing to the development of cardiovascular complications (Figure 2). Thus, vascular progenitor cells (Madonna et al., 2020) and endothelial cells (Madonna et al., 2020) can be identified as putative “vascular targets,” and cardiospheres as “cardiac targets” of anti-cancer drugs, operating as mediators for VAEs and cardiotoxicity, respectively, in patients treated with TKIs and PIs. The identification of the pathways through which these drugs directely affects the vascular and cardiac regenerative compartments may allow the development and patenting of new strategies for the cardio- and vascular protection from cancer therapies, or the development of next generation anticancer drugs avoiding targeting the vascular and cardiac progenitor cells.

## Conclusions

The recent introduction of multi-target drugs for hematological malignancies has greatly expanded treatment options for the elderly. In this group of patients, the increasing presence of comorbidities and comedications exacerbates the problem of cardiotoxicity and drug interactions, respectively. Clinical trials have demonstrated that the combination of 2 or 3 comorbidities or cardiovascular risk factors significantly increases the incidence of cardiotoxicity. In a specular way cancer sequelae itself can exert a cardiotoxic effect. For example, hypercalcemia, anemia, renal failure, amyloidosis, and bone lesions can contribute to cardiovascular complications, regardless of cardiotoxicity. In addition to co-morbidities, their co-treatments can also exacerbate cardiotoxicity by interfering with cardiac ischaemic tolerance and endogenous cardioprotective signaling pathways. Interference between co-drugs and anticancer drugs may be due to reciprocal pharmacokinetic or pharmacodynamic interactions. Senescence of cardiac and vascular progenitor cells is the Achilles heel that further contributes to the marked cardiovascular toxicity of anticancer therapies in the elderly. New generation drugs are often tested on young people and there is no specific enrollment of the elderly in clinical trials. The specific and targeted inhibition of “on-target off-tumor” effects (Madonna et al., 2020, 2021), the design of clinical trials for the elderly, and better understanding of the mechanisms driving cardiovascular toxicity, both within vascular and cardiac regenerative compartments, could represent a strategy for the treatment of cardiovascular toxicity induced by multi-target anticancer drugs in the elderly with hematological malignacies, without interfering with the antitumor effect of drugs. Future studies must develop specific monitoring systems and stratification scores for patients treated with TKI or PIs, that are able to identify the subgroups most at risk of developing the specific cardiotoxicity from TKI PIs, an aspect that is currently deficient in the cardio-oncology field.

## Author Contributions

RM: design, writing, and funding.

## Funding

This work was supported by a grant from Incyte s.r.l. to RM and funds from Ministero dell'Istruzione, Università e Ricerca Scientifica to RM (549901_2020_Madonna:Ateneo). The funders had no role in study design, data collection and analysis, the decision to publish, or preparation of the manuscript.

## Conflict of Interest

The author declares that the research was conducted in the absence of any commercial or financial relationships that could be construed as a potential conflict of interest.

## Publisher's Note

All claims expressed in this article are solely those of the authors and do not necessarily represent those of their affiliated organizations, or those of the publisher, the editors and the reviewers. Any product that may be evaluated in this article, or claim that may be made by its manufacturer, is not guaranteed or endorsed by the publisher.
